# Designing bioinspired composite reinforcement architectures via 3D magnetic printing

**DOI:** 10.1038/ncomms9641

**Published:** 2015-10-23

**Authors:** Joshua J. Martin, Brad E. Fiore, Randall M. Erb

**Affiliations:** 1 Department of Mechanical and Industrial Engineering, Northeastern University, Boston, Massachusetts 02115, USA

## Abstract

Discontinuous fibre composites represent a class of materials that are strong, lightweight and have remarkable fracture toughness. These advantages partially explain the abundance and variety of discontinuous fibre composites that have evolved in the natural world. Many natural structures out-perform the conventional synthetic counterparts due, in part, to the more elaborate reinforcement architectures that occur in natural composites. Here we present an additive manufacturing approach that combines real-time colloidal assembly with existing additive manufacturing technologies to create highly programmable discontinuous fibre composites. This technology, termed as ‘3D magnetic printing', has enabled us to recreate complex bioinspired reinforcement architectures that deliver enhanced material performance compared with monolithic structures. Further, we demonstrate that we can now design and evolve elaborate reinforcement architectures that are not found in nature, demonstrating a high level of possible customization in discontinuous fibre composites with arbitrary geometries.

## Abstract (web summary)

Superior mechanical properties in natural composites are frequently achieved by the inclusion of locally orientated reinforcing particles. Here, the authors implement this design strategy synthetically by employing a 3D magnetic printing protocol to create programmable composite architectures.

## Graphical abstract

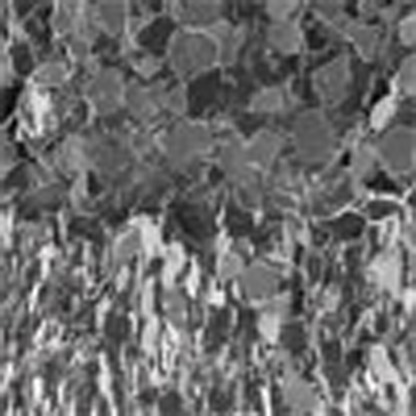

Natural composites utilize reinforcing particles exquisitely organized into complex architectures to achieve superior mechanical properties including the shells of abalones^1^, the dactyl clubs of peacock mantis shrimp^2^,^3^ and the cortical bones of mammals^4^. To grow these reinforcement architectures, biological systems invoke complex cellular and molecular processes. Such ordered, yet heterogeneous, reinforcement architectures are frequently linked to superior mechanical properties. The diversity of reinforcement architectures in natural materials far exceeds the composite design currently available in synthetic materials. Reinforcement architectures in synthetic composites are currently limited, in part, by our inability to control the local orientation of the stiff elements that comprise the reinforcement architecture. Though natural manufacturing processes are complex, novel colloidal assembly techniques and advances in additive manufacturing can be harnessed to also grow synthetic composites with similarly complex architectures.

Additive manufacturing (three-dimensional (3D) printing) offers a very effective platform for generating complex 3D architectures out of a variety of materials from polymers^5^ to metals^6^,^7^ to ceramics^8^. These structures have voxel resolution down to tens of microns and have been used to print load-bearing mechanical structures from gears to functional fuel nozzles for jet engines^9^. The printing of polymers has been accomplished mainly by extrusion-based methods for thermoplastics and stereolithography (SLA)-based photo-polymerization for both thermoplastics and thermosets^5^. 3D printing represents one of the most effective ways to manufacture customized parts with significant complexity; which explains its ubiquity in industry, academia and personal use^10^. As printed polymers are lightweight but relatively weak, 3D printing is now moving toward manufacturing fibre-reinforced polymer composites owing to their impressive strength-to-weight ratios^11^. Industrial-scale automated fibre placement printers have been developed that can print continuous strand carbon fibre-, Kevlar- and fiberglass-reinforced polymer^12^. These precision printers are currently limited to reinforcement fibres with length scales greater than millimetres and geometries larger than centimeters with orientation control limited to the *x*–*y* plane.

Arguably the greatest challenge of adapting additive manufacturing technology to discontinuous fibre-reinforced composites is the ability to orient reinforcing fibres during the printing process^13^,^14^. Fibres aligned parallel to the predominant stresses reinforce the encompassing matrix, while orthogonal fibres act as defects, weakening the polymer matrix^15^,^16^. Randomized fibres (standard for systems without control) perform between these two extremes and negligibly affect composite strength while severely sacrificing ductility^17^. Dimas *et al*.^18^ provided a first attempt at using 3D printing to create tough composites inspired by bone to achieve a toughness modulus over 20 times that of its constituents. In this work the length scale of the hard and soft phases are in the range of several millimetres. In a movement toward micro-reinforcement, Compton *et al*.^19^ developed an epoxy-based ink that allows for the 3D printing of lightweight cellular composites with high aspect ratio microfibers that are aligned by the shear forces generated during the printing process. Though fibre orientation here is only in the direction of the flow, which limits design complexity, the Young's moduli of the printed structures are 10 times greater than existing commercially available 3D-printed polymers. With greater precision over the reinforcing architecture, more complex and functional materials created via additive manufacturing will find application in a wide range of engineering disciplines.

## Results

### 3D Magnetic Printed Composites

Here we detail a distinctly different approach termed as 3D magnetic printing that orients anisotropic reinforcing particles during the printing of composites using magnetic fields. We can produce elegant reinforcement architectures of ceramic microparticles that exhibit feature sizes of 90 μm. These architectures enable composite materials that exhibit increased stiffness, strength and hardness properties. This method is robust, low cost, scalable, sustainable and will enable an entirely new class of strong, lightweight composite prototypes with programmable properties.

**Figure 1:**
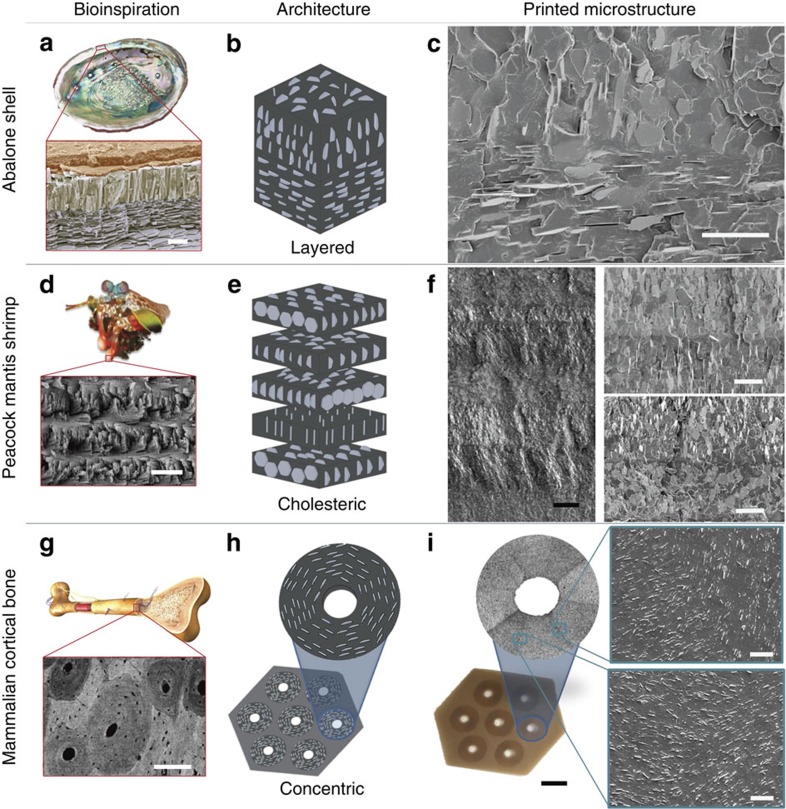
Bioinspired composites with microstructured architectures can be recreated with 3D magnetic printing. (**a**) The *Haliotidae sp.* abalone shell exhibits a layered structure of calcite prisms topping in-plane aragonite platelets (nacre). Reprinted from ref. 27 (reproduced with permission from Wiley-VCH). This architecture is (**b**) simplified and (**c**) 3D magnetic printed. (**d**) The dactyl club of the peacock mantis shrimp exhibits a cholesteric architecture of mineralized chitin fibres^2^,^3^. Reprinted from ref. 3 (reproduced with permission from Elsevier). This architecture is (**e**) simplified and (**f**) 3D magnetic printed. (**g**) The mammalian cortical bone exhibits concentric plywood structures of lamellae-reinforced osteons^4^. Reprinted from ref. 28 (reproduced with permission from Elsevier). This architecture is (**h**) simplified and (**i**) 3D magnetic printed. All printed microstructures are acrylate-urethane co-polymers reinforced by 15 volume percent alumina platelets. Scale bar, 5 μm in **a**; 25 μm in **c**; 15 μm in **d**; 50 μm (black) and 20 μm (white) in **f**; 200 μm in **g**; and 5 mm (black) and 25 μm (white) in **i**.

To demonstrate the capability of this technique we recreated choice reinforcement architectures exhibited by biological discontinuous fibre composite systems including the osteon structures within mammalian cortical bone, the layered nacreous shell of abalones and the cholesteric reinforced dactyl club of the peacock mantis shrimp (Fig. 1). In each case, the specific orientation of the reinforcing elements is believed to contribute to the outstanding composite properties including enhanced strength, high stiffness and extraordinary toughness. The reinforcement architecture in each natural composite is conveyed in terms of a simplified microstructure translated to platelet reinforcement. These microstructures are imported into our 3D magnetic printing framework to create bulk composites with fine-tuned, bioinspired microstructural design.

### 3D Magnetic-Printing Process

**Figure 2:**
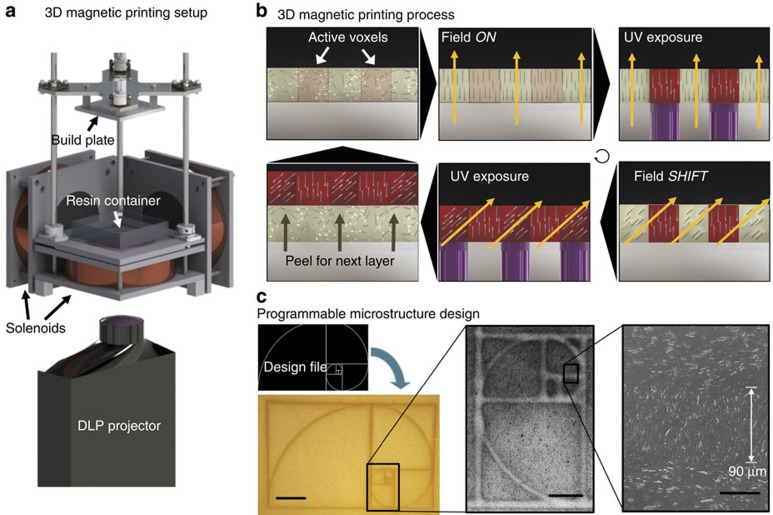
The 3D magnetic printing process. (**a**) The 3D magnetic-printer setup uses a digital light processor (DLP) to photo-polymerize resin with ultra-violet (UV) while a magnetic field is simultaneously applied via electromagnetic solenoids. (**b**) The 3D magnetic printing process systematically aligns and selectively polymerizes groupings of voxels programmed to have specific reinforcement orientation within each layer of printed material based upon a shifting field. The build plate peels after a layer is complete to print additional layers. (**c**) With 3D magnetic printing, detailed reinforcement micro-architectures can be printed from design files including this example of the golden rectangle which exhibits clear feature sizes as low as 90 μm. Scale bar, 2 mm, 500 and 50 μm in **c** from left to right.

We have created a SLA-based 3D printing framework, termed as 3D magnetic printing, that is capable of printing dense ceramic/polymer composites in which the direction of the ceramic-reinforcing particles can be finely tuned within each individual voxel of printed material (Fig. 2a). To enable the orientation of the ceramic-reinforcing particles, we employ magnetic-labeling techniques to coat traditionally nonmagnetic-reinforcing materials such as high-strength alumina, silica and calcium phosphate with nominal amounts of iron oxide nanoparticles^20^,^21^. 3D magnetic printing can produce a wide variety of novel composite micro-architectures with clearly printed features down to 90 μm in size exhibiting unique reinforcement. The 3D magnetic printing process is necessarily hierarchical with the magnetic nanoparticles (∼10 nm) responding to external fields to orient the ceramic microparticles (∼10 um) during the print that work to reinforce each printed polymer voxel (∼100 um) resulting in a complex macroscopic composite architecture (∼10 cm).

Standard SLA-printing processes involve ultra-violet (UV)-polymerization of an entire set of active voxels simultaneously within one layer of uncured polymer. Instead, the 3D magnetic-printing process incorporates additional steps to orient the ceramic microparticles during the print as shown in Fig. 2b. First, voxels within each print layer are separated into groupings of similar reinforcement orientation. Second, prior to polymerizing the first active grouping of voxels, a rotating magnetic field is applied to align the long axes of the reinforcing microparticles^22^. Alignment time in the polymer precursor suspension is ∼12 s depending on the applied field strength, solution viscosity, nanoparticle coverage and the microparticle geometry (see Supplementary Discussion and Supplementary Fig. 1). Third, a digital light projector (DLP) polymerizes this active grouping of voxels simultaneously in 10 s consolidating the structure and fixing the orientation of the reinforcement with UV light. Fourth, the next set of active voxels is aligned in a shifted magnetic field to set a new orientation. Fifth, these active voxels are then UV-polymerized. These last two steps are repeated for each unique reinforcement orientation that is to be incorporated into the composite layer. Finally, the cured polymer is peeled from the base as the build plate is lifted. This brings in uncured ink into the printing area for the next layer to be printed.

To demonstrate the robustness of this method, a variety of designs were implemented to define the reinforcement micro-architecture including the bioinspired microstructures shown in Fig. 1 and the geometric pattern of the golden rectangle shown in Fig. 2c that exhibits feature sizes as low as 90 μm. Though this technique is not material specific, the micro-architectures presented here are solid printed composite blocks of urethane/acrylate copolymer reinforced by 15 volume percent of alumina microplatelets with site-specific orientation. As an example, the printed composite in Fig. 2c consists of a binary reinforcement pattern (microplatelet angles changing between *θ*=0° and *θ*=90°) in which 300 by 185 voxels are shown. The orientation of the alumina microparticles results in an optical change to the composite surface. Reinforcement oriented in-plane scatter more light and appear whiter while reinforcement oriented out-of-plane absorb more light and appear darker. However, a key aspect of this process is that, except for the local orientation of the reinforcement particles, completely homogeneous materials are being produced. This printing process required four sequential steps including (i) aligning the platelets *θ*=0° with an in-plane rotating magnetic field, (ii) UV-curing the voxels programmed to have *θ*=0° reinforcement with the DLP, (iii) aligning the remaining platelets *θ*=90° with an out-of-plane rotating magnetic field and (iv) UV-curing the voxels programmed to have *θ*=90° reinforcement with the DLP. These four steps took 2 min to fabricate an entire composite layer. Analysis of the underlying reinforcement microstructures with SEM revealed high levels of microparticle alignment in the final composites. See Supplementary Figs 2 to 13 for additional examples.

### 3D Micro-Architectural Design

**Figure 3:**
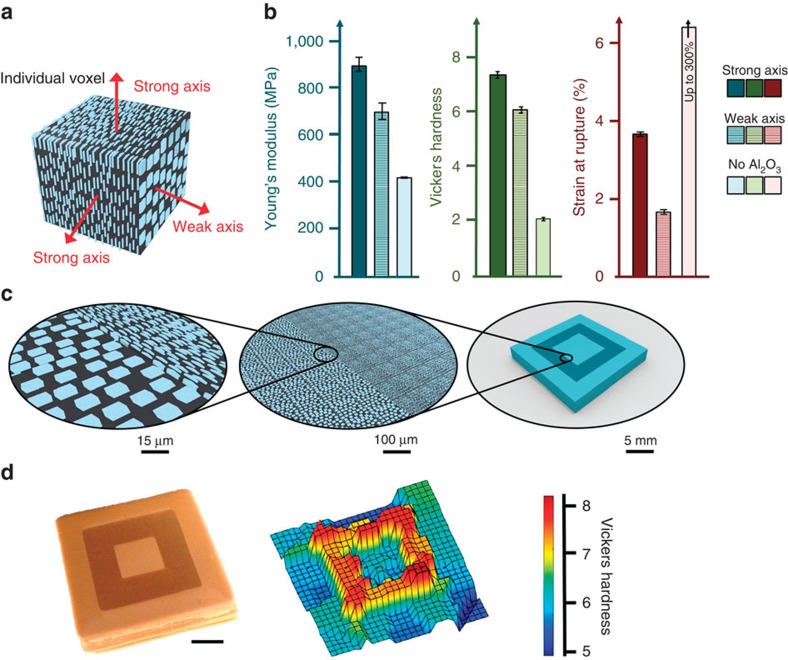
Mechanical properties of 3D magnetic printed composites. (**a**) Schematic of an individual voxel indicating the two strong and one weak axes of a polymer voxel containing fully oriented ceramic microplatelets. (**b**) Tensile tests were conducted on printed composites with monolithic reinforcement orientation. Printed composites with voxels containing ceramic microparticles aligned parallel to the principal stresses exhibited higher stiffness (+29%), higher hardness (+23%) and higher strain at rupture (+100%) as compared with composites with voxels exhibiting perpendicular alignment. (**c**) The hierarchy of a 3D magnetic-printed block with a concentric square pattern is schematically shown. (**d**) The block is produced with 3D magnetic printing (the block is 2.2 × 2.2 cm and 3 mm thick) and subjected to hardness mapping. Scale bar, 4 mm in **d**.

3D magnetic printing enables an entirely new class of strong, lightweight composites. We envision a manufacturing process that starts with a user inputting a digital geometry via computer aided design (CAD) software or an imaging device (for example, 3D laser scanner), applying finite element mechanical analysis on the digital geometry to find an optimized reinforcement architecture and 3D printing the optimum architecture by orienting reinforcement in each voxel. The ability to tune reinforcement architectures provides wide programmability to the stiffness, strength, toughness and multi-functionality of composite materials. To investigate these claims, we characterized monolithic blocks of 3D magnetic printed composites (that is, all voxels having the same orientation) with tensile tests to measure the material properties along each axes (Fig. 3a,b). Axes with reinforcement aligned parallel to the principal stresses (strong axis) exhibited both enhanced stiffness and hardness, which is the expected result from composite theory. In addition, strong-axis composites had a strain at rupture twofold larger than weak-axis composites (4% versus 2%, both much less than the 300% of the pure matrix). To validate that these material properties are maintained within each voxel in a complex printed structure, a micro-architecture was designed and subjected to hardness mapping to measure local material properties (Fig. 3c). Hardness mapping has been used to demonstrate the detailed programmability of composites in the natural world especially in graded structures that have heterogeneous hardness^23^,^24^. Voxels with reinforcement oriented out-of-plane demonstrate a significant increase in out-of-plane hardness relative to voxels with in-plane reinforcement.

**Figure 4:**
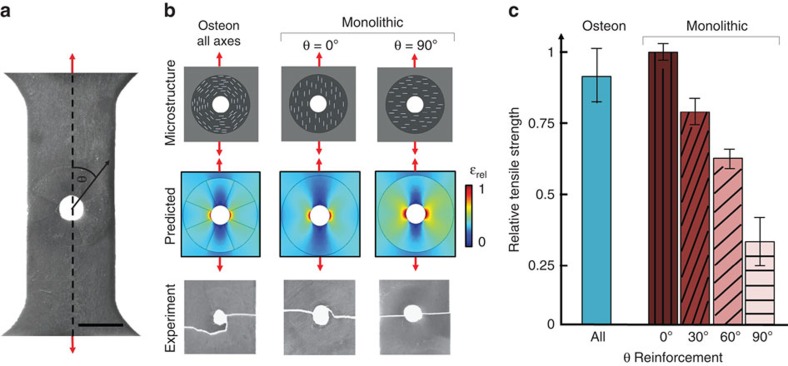
Mechanical analysis of printed composites with circular defects. (**a**) Samples with circular defects are 3D magnetic printed with programmable reinforcement architectures including ‘osteon-inspired' microstructures with concentric reinforcement orientation and ‘monolithic' microstructures as shown in **b**. (**b**) ‘Osteon-inspired' microstructures are predicted to exhibit less relative strain (**ɛ**_rel_) compared with misaligned 
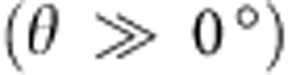
 ‘monolithic' microstructures. (**c**) Tensile tests of printed composites with circular defects show that ‘osteon-inspired' architectures out-perform all but the perfectly aligned ‘monolithic' sample. As the ‘osteon-inspired' architecture is symmetric, the load can be applied at any angle relative to the microstructure to obtain similar performance. Scale bar, 5 mm in **a**.

In addition, microstructure is believed to play a role in dictating fracture mechanics especially in heterogeneous natural composites such as cortical bone. Despite the presence of many cylindrical defects containing vasculature (Harversian Canals), cortical bone maintains impressive mechanical properties in part due to the concentric plywood microstructures of lamellae-reinforced osteons^4^. With 3D magnetic printing, reinforced composite dogbones with various architectures surrounding large circular defects were printed. Tested architectures included ‘osteon-inspired' structures with reinforcement concentrically aligned around the defect and ‘monolithic' structures with fully-aligned reinforcement at various angles (Fig. 4a,b, see Supplementary Table 1). These unique architectures were modelled with finite element analysis to observe anticipated strains surrounding these circular defects. The strain was plotted as a relative strain, *ɛ*_*rel*_, that reflects the ratio between the local principle strain with the maximum principle strain in all microstructures, which occurred at the edge of the circular defect in the monolithic structure with *θ*=90°, (see Supplementary Methods for modelling details). The ‘osteon-inspired' architecture characterized by concentric orientation of the reinforcing platelets exhibited the lowest strain concentration and the maximum average tensile strength except for the 0° monolithic microstructure (Fig. 4b, c). However, since the ‘osteon-inspired' architecture is symmetric, the load can be applied at any angle relative to the microstructure to obtain similarly high performance. Within the framework of 3D magnetic printing, the composite architecture can be optimized between the digital generation of the geometry and the actual printing of the material. This optimization of the architecture can be implemented with the help of FEA analysis to evolve the composite architecture toward enhanced performance metrics such as higher strength and stiffness.

### Crack Steering via 3D Magnetic Printing

Detailed analysis of the synthetic osteon structures revealed apparent differences in the fracture behaviour between both the ‘osteon-inspired' and the 0° monolithic versus the 90° monolithic. The 90° monolithic structure tended to show a straight, fast crack propagation related to cleavage^25^ while the ‘osteon-inspired' and 0° monolithic architecture tended to exhibit deflection during the propagation of the crack leading to a tortuous crack path. These results indicated that 3D magnetic printing enables the creation of micro-architectures that increase deflection in the crack path (see Supplementary Fig. 9). Such crack deviation has often been associated with increased fracture toughness especially in human bone^16^ and nacre^26^.

**Figure 5:**
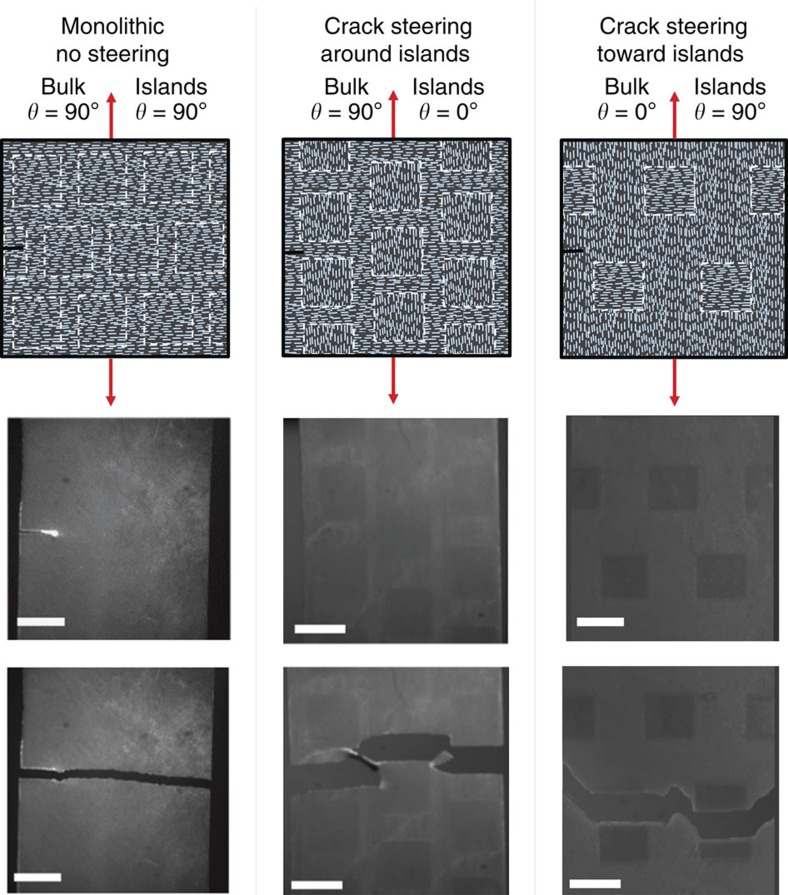
Crack steering with 3D magnetic printed architectures. 3D magnetic-printed architectures with islands that match and contrast the reinforcement orientation of the bulk film. Intricate micro-architectures are found to be capable of crack steering (lengthening the crack). Scale bar, 4 mm.

Crack propagation pathways can be further tuned by creating architectures with 3D magnetic printing that are designed to exaggerate deviation in the crack path. Figure 5 shows examples of printed structures in which 4 × 4 mm square islands of varying reinforcement orientation were printed in a first step and an encompassing monolithic background was printed in a second step to make a solid composite. When the island orientation matched the background, a monolithic structure was produced and crack behaviour matched that expected of monolithic composite materials. However, when the island orientation contrasted the background orientation, the crack was steered through the material. In the case where the island orientation was perpendicular to the principal crack direction, the islands acted as strong points and repelled the crack tip. In the case where the island orientation was parallel to the principal crack direction, the islands acted as weak points and attracted the crack tip. Crack steering provides a possible control toggle over fracture-toughening mechanisms in composites microstructures. For example, this property can be used to program failure mechanisms in a material that would deflect cracks away from crucial areas of a structure.

## Discussion

Overall, 3D magnetic printing shows great promise as a new manufacturing tool to design the micro-architecture of reinforced composites to maximize material properties. Print speeds are reasonable (1 min per programmed reinforcement orientation per layer) but can be further improved. We have used this technique to recreate bioinspired composite micro-architectures from ‘concentric' structures of the osteon to the ‘layered' structures of nacre to the ‘cholesteric' structures of the peacock mantis shrimp. Printed composite materials demonstrate clear mechanical dependence upon their underlying micro-architecture through hardness mapping and tensile testing. Finally, the capacity to tune the fracture of these printed composites is demonstrated by steering cracks in a fracturing sample.

## Methods

### Magnetic-Labeling of Reinforcing Particles

Al_2_O_3_ (alumina) particles were kindly supplied by Antaria (Australia). The alumina particles are in platelet form with an average diameter of 7.5 um and an average thickness of 350 nm. This provides an aspect ratio of around 21. To magnetize the alumina platelets, 375 μl of superparamagnetic iron oxide nanoparticles (SPIONS, EMG-705, 3.9% vol Fe_3_O_4_, Ferrotec, Bedford, NH, USA) was titrated with 10 g of Al_2_O_3_ in 200 ml of deionized water to ensure a homogenous coating of the microparticles. The mixture was stirred overnight using a magnetic stir bar. A negatively charged ligand-coating on the iron oxide allows the nanoparticles to electrostatically adsorb to the surface of the alumina particles. The particles are subsequently, filtered and dried. Once drying is complete, the magnetized alumina particles (m-Al_2_O_3_) were added to the photopolymer at desired volume fractions.

### Preparation of Photopolymer-Ceramic Composite Resin

UV-sensitive resin was made by first mixing aliphatic urethane diacrylate (Ebecryl 230) and isobornyl acrylate (IBOA, Sigma) in a 1:3 ratio by weight. Photo-initiators (phenylbis(2,4,6-trimethyl-benzoyl)phosphine oxide 97%, 1-Hydroxycyclohexyl phenyl ketone 99%) were added at 2% and 3% weight, and stirred overnight. The viscosity of the polymer blend was measured using an Ubbelohde viscometer (SimpleVIS, size 2C) and was found to be 140 mPa s. Desired volume fractions of m-Al_2_O_3_ were added to the resin and were sonicated in volumes of 30 ml using a microtip sonifier (Branson 250, 20% duty cycle, 40 W output for 10 min) to ensure sufficient exfoliation. Finally, the resulting mixture was degassed to prevent bubbles from causing defects during the printing process.

### 3D Magnetic Printing Device

The custom stereolithographic 3D printer used in this work utilizes an open-source software (Creation Workshop) to control a DLP projector (ViewSonic PJD7820hd) and two NEMA-17 stepper motors for the *z*-axis. The software's native slicing engine converts ‘.stl' files to a series of high-resolution vector files that are used to polymerize each cross section. The source files for this device are not limited to ‘.stl' files, and can also be generated directly from a ‘.jpeg, .tiff,' and others. The printer's frame was purchased from mUVe 3D and modified to allow for the application of magnetic fields by either rotating a rare-earth magnet or using computer-controlled solenoids (4.5 in inner diameter, 2 in thick, 6 in outer diameter copper coil, Endicott Coil).

## Additional information

**How to cite this article:** Martin, J. J. *et al*. Designing bioinspired composite reinforcement architectures via 3D magnetic printing. *Nat. Commun.* 6:8641 doi: 10.1038/ncomms9641 (2015).

## Supplementary Material

Supplementary Information Supplementary Figures 1-13, Supplementary Table 1, Supplementary Discussion, Supplementary Methods and Supplementary References
