# Supplementary material for: Designing bioinspired composite reinforcement architectures via 3D magnetic printing
Source: Nat Commun. 2015 Oct 23;6:8641. doi: 10.1038/ncomms9641 (PMC4639893; doi:10.1038/ncomms9641)
Supplement: Supplementary Information — Supplementary Figures 1-13, Supplementary Table 1, Supplementary Discussion, Supplementary Methods and Supplementary References [file ncomms9641-s1.pdf]

Supplementary Figures:

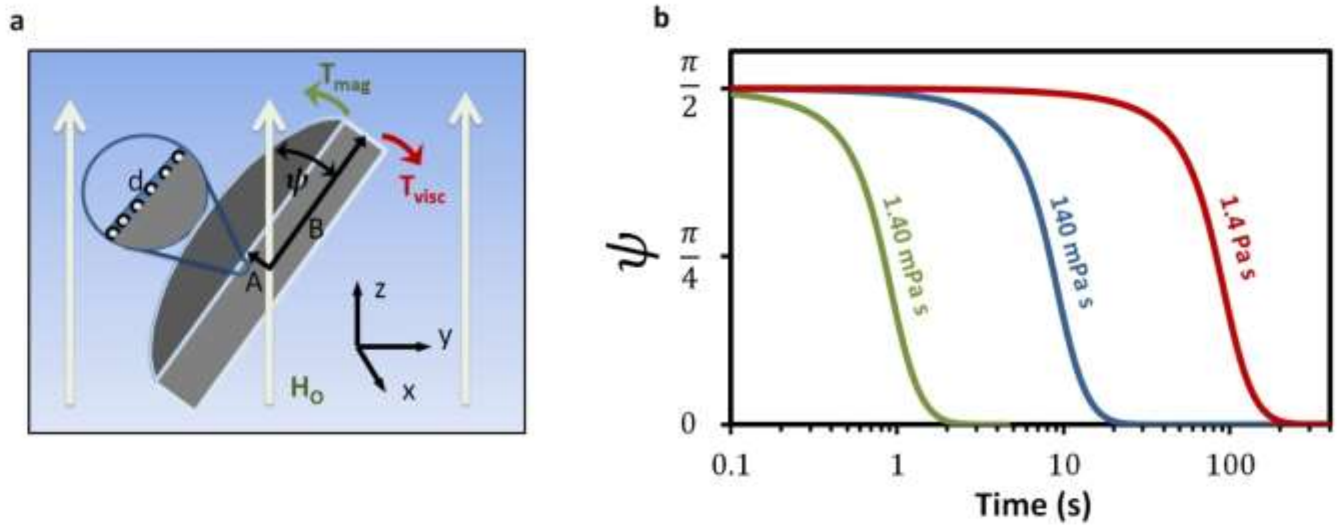

Supplementary Figure 1. (a) *The magnetics diagram shows the parameters used in determining the alignment time necessary for a magnetically labeled platelet. (**b**) The angle versus time plots show the response of the platelet in different mediums in the presence of a 400 Oe (0.04T) field.*

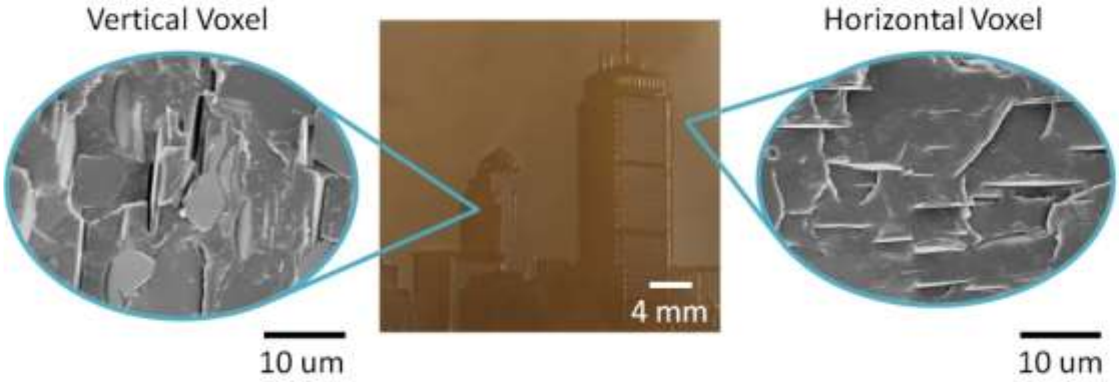

Supplementary Figure 2. *The Boston skyline was printed using out-of-plane voxels (darker regions) and in plane-voxels (lighter regions). Cross-sectional SEM images of both the in-plane and out-of-plane orientation clearly demonstrate particle orientation.*

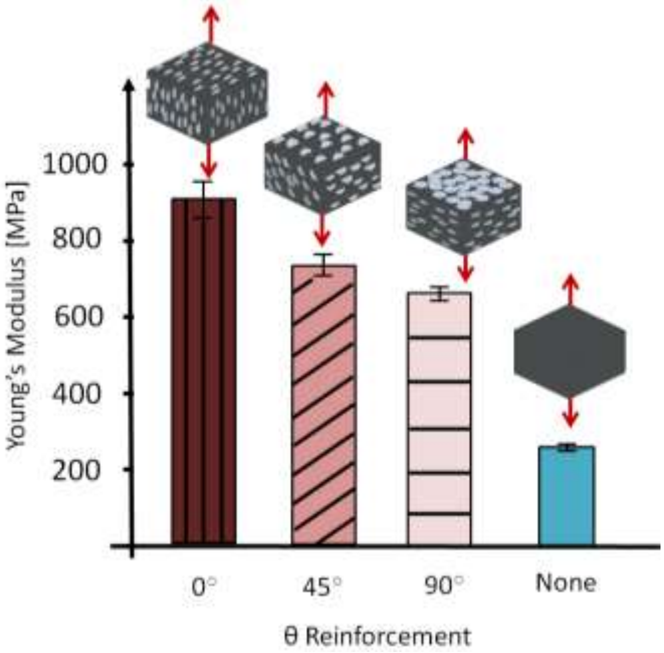

Supplementary Figure 3. *Young's modulus vs reinforcement orientation for polymer-ceramic composites at 15% volume for reinforcing particles at 0°, 45°, and 90° degrees and without reinforcement.*

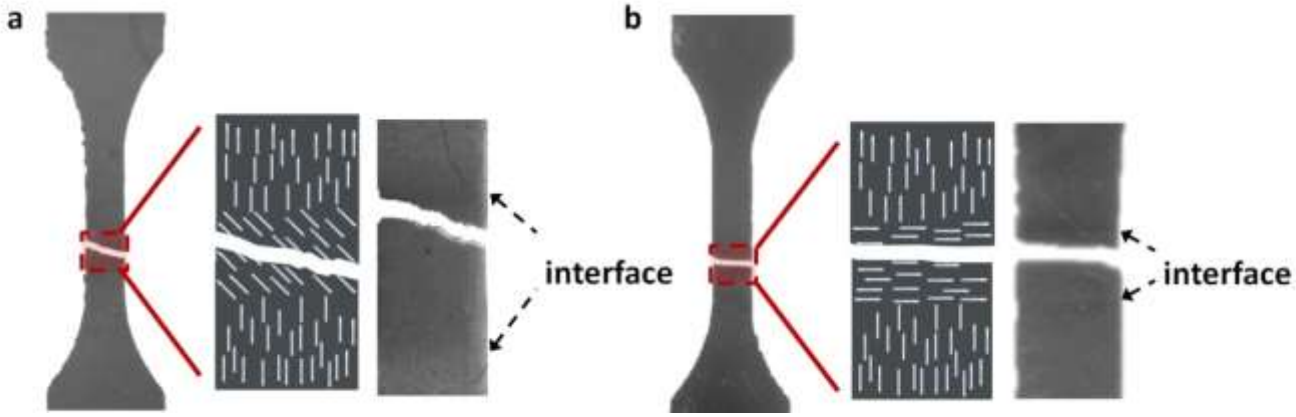

Supplementary Figure 4. *Tensile specimens reinforced with a designed weakness exhibit failure not at the interface between phases, but within the weaker area. (**a**) The printed tensile specimen exhibits a center region reinforced at 45 degrees to the axis of the applied load. (**b**) Exhibits a specimen with reinforcement that is perpendicular to the applied load.*

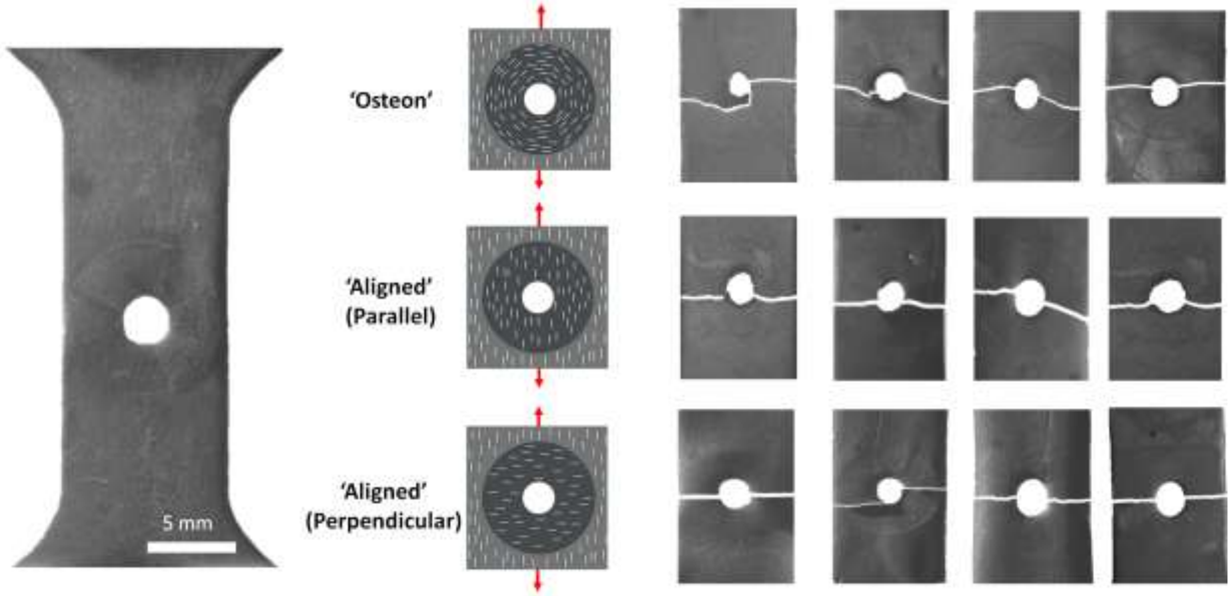

Supplementary Figure 5. Tensile specimens with circular defects were printed with different reinforcing microstructures: osteon-inspired (concentric), parallel to the applied load, and perpendicular to the applied load.

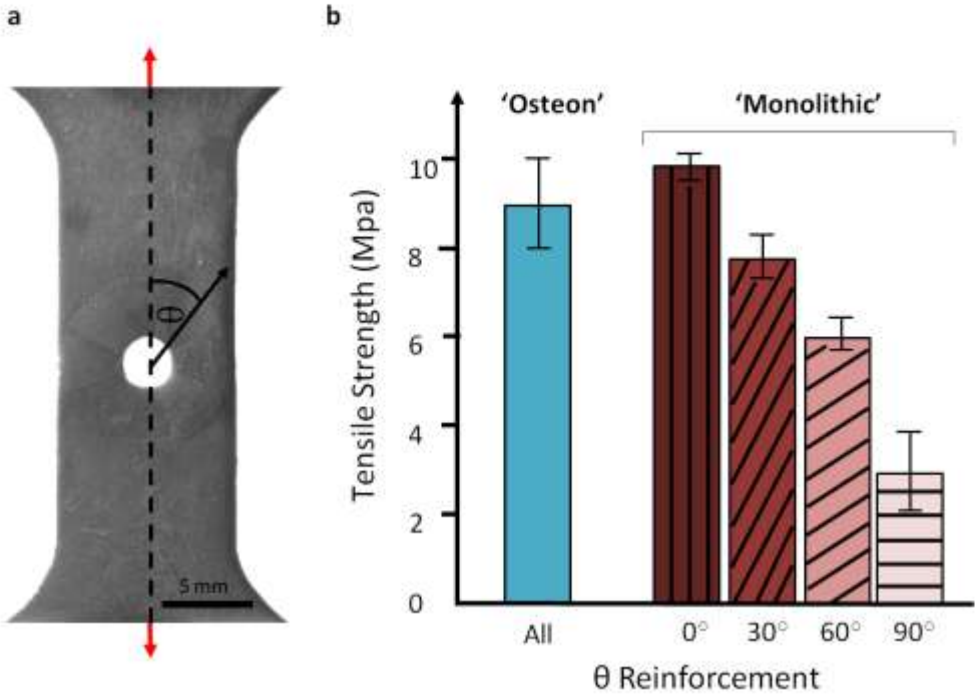

Supplementary Figure 6. (a,b) Tensile strengths of specimens with centered defects were compared with different reinforcing architectures: osteon inspired (all), parallel to the applied load (0 degrees), 30 degrees to the applied load, 60 degrees to the applied load, and perpendicular to the applied load (90 degrees). Tensile strengths are normalized according to each specimens' cross-section area at the point of fracture.

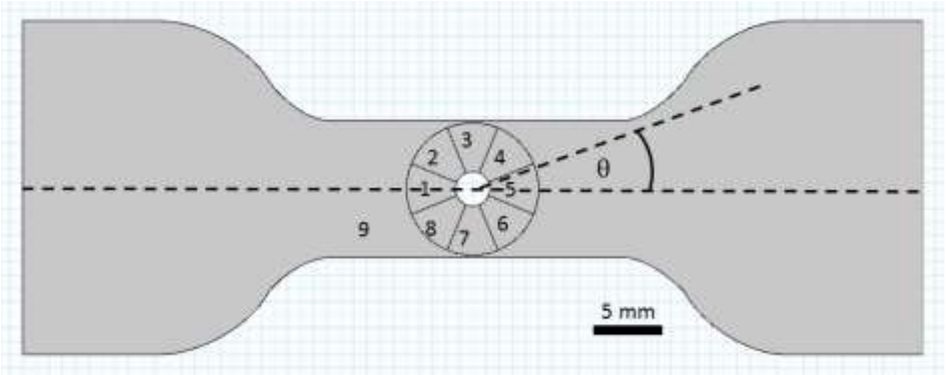

Supplementary Figure 7. Geometric model used for the numerical evaluation of dogbone samples with circular defects.

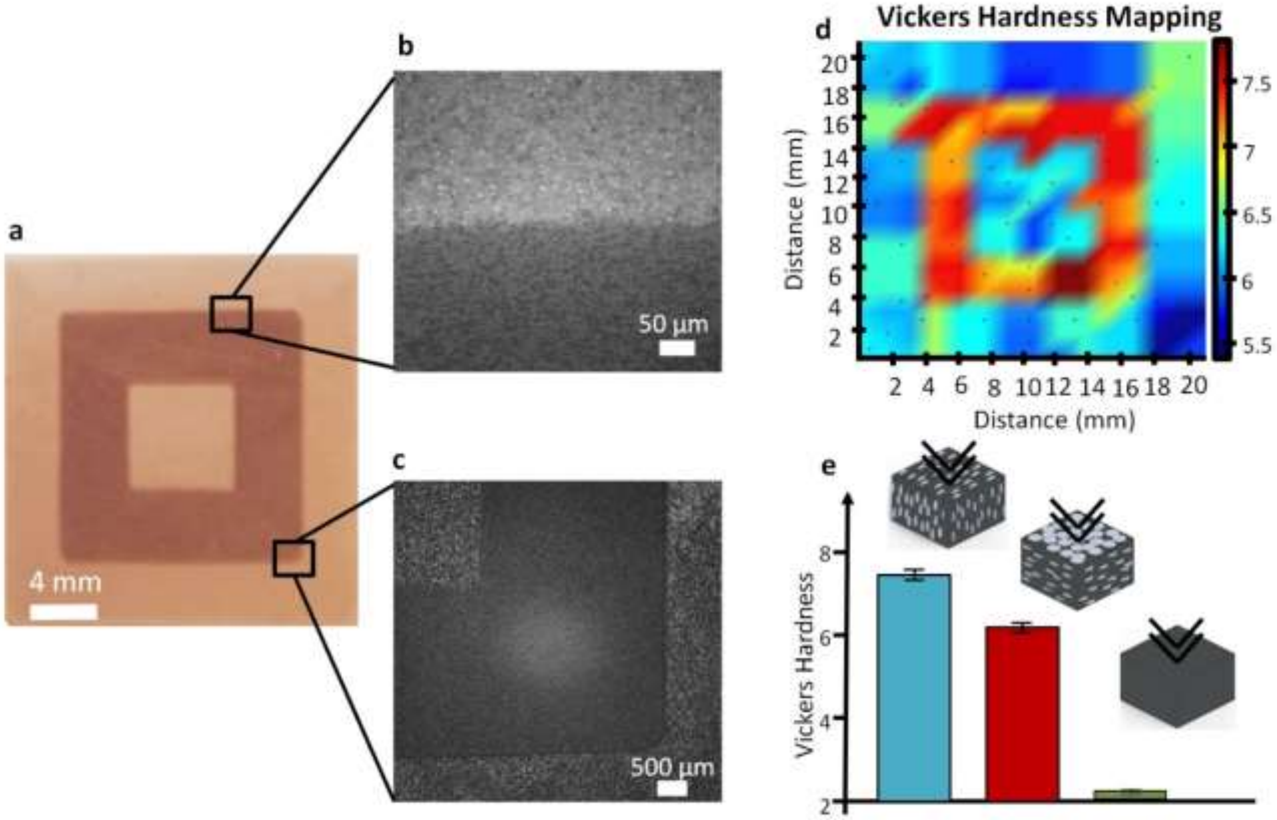

Supplementary Figure 8. a-e, *Optical microscope images of a 3D printed specimen with an orthogonal-rectilinear pattern reveal stark contrasts in orientation. Surface plots of the hardness mapping demonstrate distinct changes in mechanical properties as a function of orientation.*

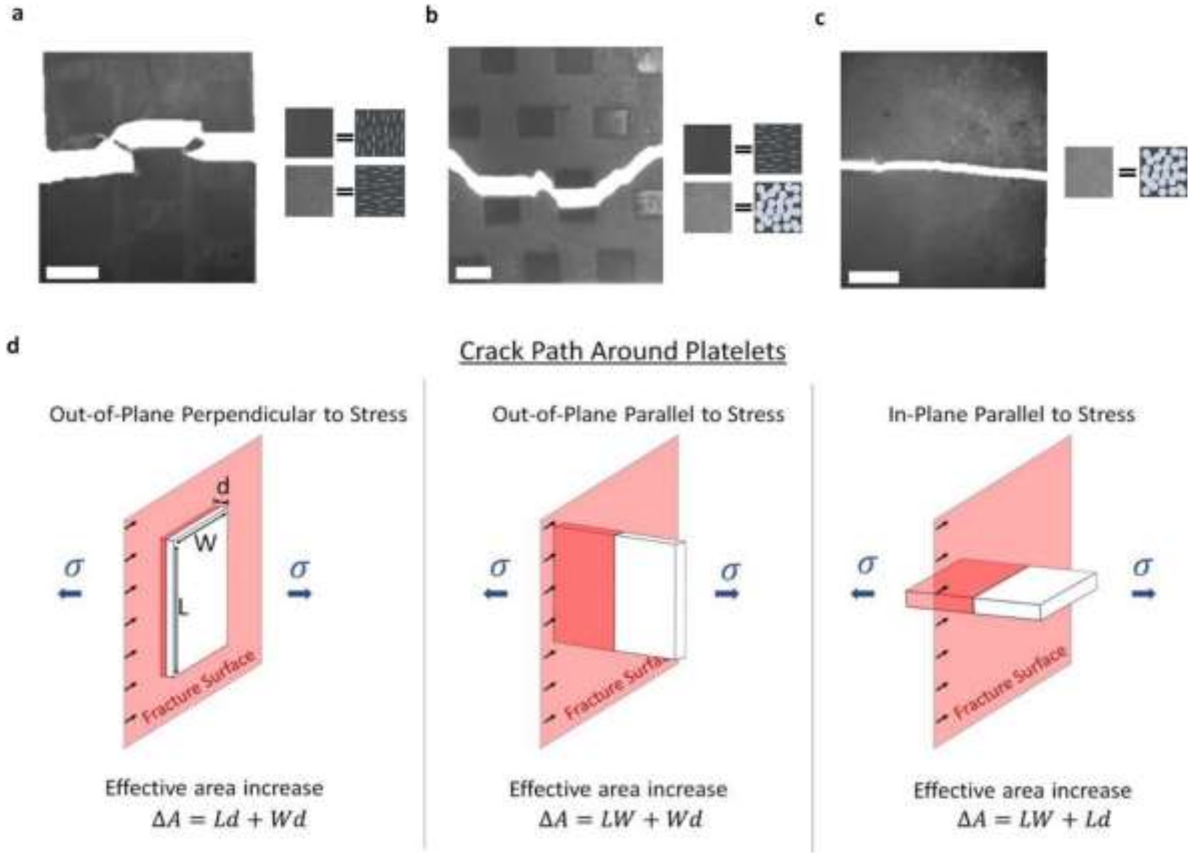

Supplementary Figure 9. *Tensile specimens were printed with various reinforcement architectures are notched prior to testing. (**a**) A sample containing two orthogonal orientations that were slightly offset. (**b**) A sample in containing islands reinforced parallel to the crack direction. (**c**) The sample contains a monolithic reinforcement structure. (**d**) The behavior of crack path around reinforcing platelets is exhibited for different orientations as a function of interfacial surface area. All scale bars are 4 mm.*

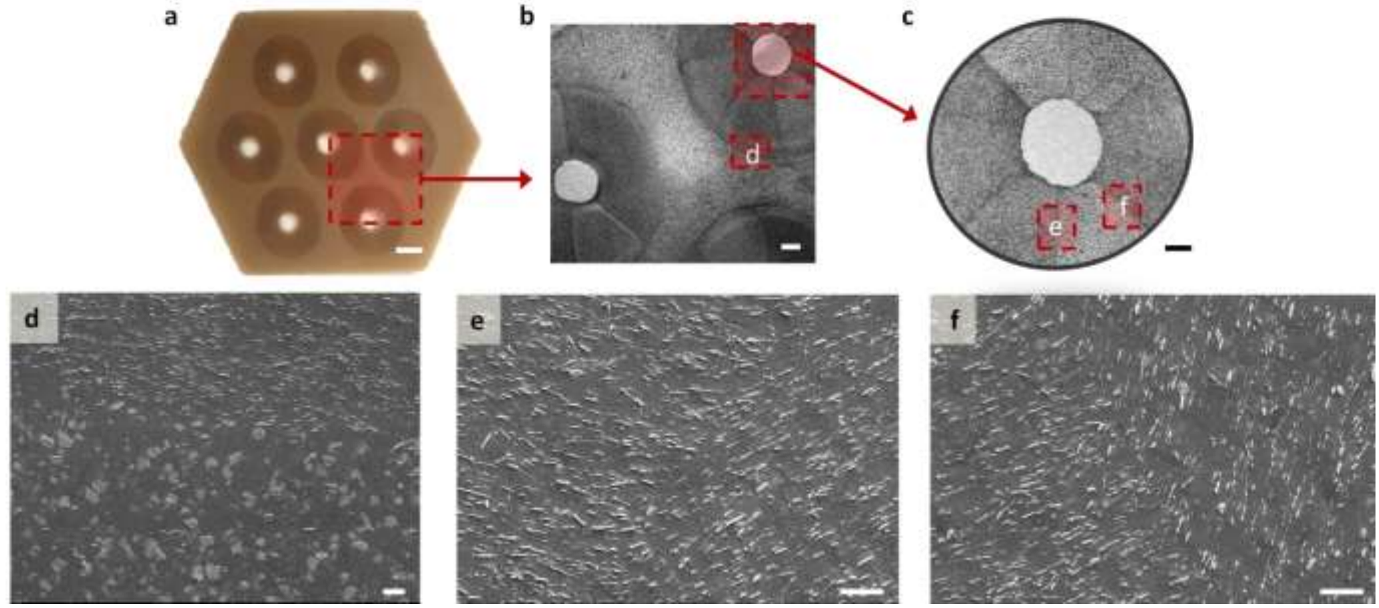

Supplementary Figure 10. *The hexagonal array in a. was 3D printed and viewed under an optical microscope to observe the geometry of the system. Platelet orientation can be viewed in the SEM images, corresponding to specific regions of the print. The scale bar is 2 mm in a, 500 µm in b and c, and 25 µm in d-f.*

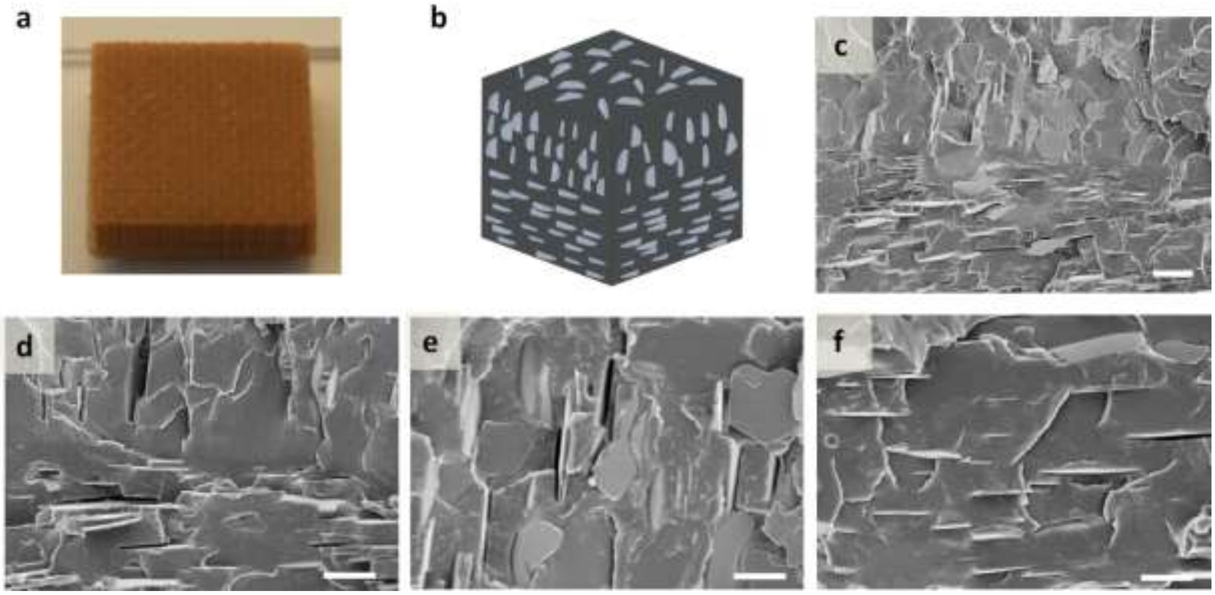

Supplementary Figure 11. *(**a**) The 3D printed block with (**b**) a layered microstructure was (**c**) freeze fractured and observed via SEM. (**d-f**) The cross-sectional images reveal a neatly ordered reinforcement structure and a strongly interconnected and coherent interface between each phase. Scale bars are 500 µm in a, 10 µm in c-d, and 5 µm in e-f.*

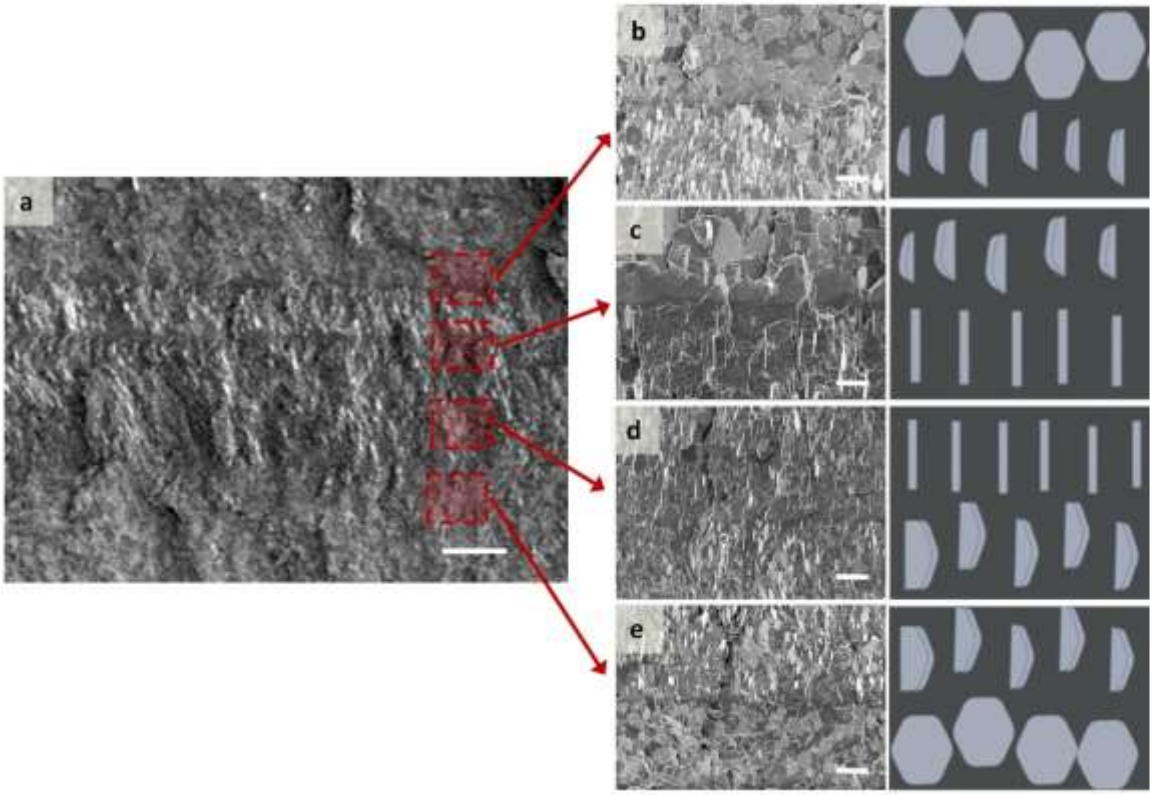

Supplementary Figure 12. *(**a-e**) The 3D printed block was freeze fractured and observed via SEM. The cross-sectional images reveal cholesteric reinforcement structure as well as a conformal interface between each phase. Scale bars are 100 µm in a, 25 µm d, and 50 µm in b, c and e.*

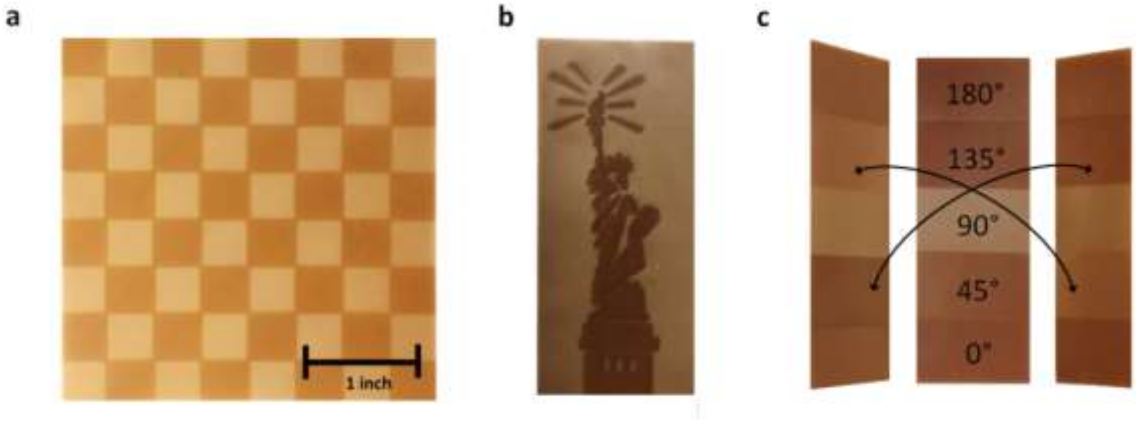

Supplementary Figure 13. *(**a**) A chessboard printed in-plane (lighter regions) and out-of-plane (darker regions) exhibits the contrast difference at 5% volume fraction particles. (**b**) Higher volume fractions (10%) lead to a similar effect on composite color. (**c**) When particles are printed at various angles, tilting the sample can result in a color-shift effect.*

Supplementary Tables:

Supplementary Table 1. The architectures are listed here. The regions are defined according to the geometry of Supplementary Figure 7.

Region:	**1**	**2**	**3**	**4**	**5**	**6**	**7**	**8**	**9**
'Osteon-inspired'	$\theta = 90°$	$\theta = 45°$	$\theta = 0°$	$\theta = 315°$	$\theta = 270°$	$\theta = 225°$	$\theta = 180°$	$\theta = 135°$	$\theta = 0°$
0° Architecture	$\theta = 0°$	$\theta = 0°$	$\theta = 0°$	$\theta = 0°$	$\theta = 0°$	$\theta = 0°$	$\theta = 0°$	$\theta = 0°$	$\theta = 0°$
30° Architecture	$\theta = 30°$	$\theta = 30°$	$\theta = 30°$	$\theta = 30°$	$\theta = 30°$	$\theta = 30°$	$\theta = 30°$	$\theta = 30°$	$\theta = 0°$
60° Architecture	$\theta = 60°$	$\theta = 60°$	$\theta = 60°$	$\theta = 60°$	$\theta = 60°$	$\theta = 60°$	$\theta = 60°$	$\theta = 60°$	$\theta = 0°$
90° Architecture	$\theta = 90°$	$\theta = 90°$	$\theta = 90°$	$\theta = 90°$	$\theta = 90°$	$\theta = 90°$	$\theta = 90°$	$\theta = 90°$	$\theta = 0°$

Supplementary Discussion:

Super-paramagnetic response of labeled particles. When a magnetic field is applied, the platelet will experience a magnetic torque that works to align one of the long axes of the platelet with the field direction. The SPIONs that coat the m-Al_2O_3 microparticles are attached to the outside of the platelets in positions that maximize the magnetic torque they can apply. These SPIONs create a discontinuous magnetic shell around the particles that can be approximated as an effectively continuous magnetic shell with a decreased magnetic susceptibility, χ_{ps}, according to the actual surface coverage of the SPIONs, ϕ, as $\chi_{ps} = \chi_{SPIONs}\phi$. By interpreting the alumina platelets and the SPION shells as oblate ellipsoids and oblate ellipsoidal shells, respectively, analytic expressions can be used to describe the magnetic energy of these systems and, thus, the resultant magnetic torques. The magnetic torque experienced by an oblate ellipsoidal shell due to an applied magnetic field, H_o, can be described as [1]:

$$T_{mag} = \frac{2\pi\mu_o\chi_{ps}^2}{3(\chi_{ps}+1)}[(A+d)(B+d)^2 - AB^2]H_o^2 sin\psi cos\psi \quad (1)$$

Here μ_o is the permeability of free space ($\mu_o = 4\pi \cdot 10^{-7}$, units of [$NA^{-2}$]), χ_{ps} is the volume susceptibility of the magnetic shell (dimensionless), H_o is the external magnetic field (units of [Am^{-1}]), and ψ is the angle between the long axis of the particle and the axis of the applied magnetic field. A and B are the lengths of the short and long axes of the platelets, respectively, and d is the diameter of the SPIONs.

This magnetic torque working to align the platelets is counteracted by a hydrodynamic torque generated by the viscous drag a platelet experiences as it rotates in the fluid. This viscous torque can be solved using Perrin friction factors (f/f_o) for ellipsoidal particles and can be expressed by [1]:

$$T_{visc} = -6\eta V(f/f_o)\left(\frac{d\psi}{dt}\right) \quad (2)$$

where V is the volume of the platelet and η is the viscosity of the surrounding fluid. Disregarding Brownian motion, which is small for these microparticle systems, the net torque can be used T_{net} to calculate the angular acceleration of the platelets under an applied field as:

$$\frac{d^2\psi}{dt^2} = \frac{T_{net}}{I} = \frac{T_{mag}+T_{visc}}{m(A^2+B^2)/5} \qquad (3)$$

Here m is the mass of the particle and I is the moment of inertia of an ellipsoid rotating around one of its long axes. Numerical solutions of this second-order differential equation were used to estimate the required time for orientation during the printing process (**Supplementary Figure 1**). The numerical solution was obtained with MatLab for an applied field of 400 Oe (0.04T) at the experimental viscosities of 1.40 mPa s, 140 mPa s, and 1400 mPa s. 140 mPa s corresponds to the precursor polymer suspension used in this work. For the alumina particles used in the study, the small axis is $A = 187.5\ nm$, the long axis is $B = 3.75\ \mu m$, and the thickness of the magnetic shell is $d = 12\ nm$.

Supplementary Methods:

3D Magnetic Printing Process. The printing process (**Figure 2b** in the main text) begins by applying magnetic fields to orient the reinforcing particles within the resin container. Rotating magnetic fields (3 Hz) are applied with a magnitude of 400 Oe to achieve the desired alignment architecture. Once the proper alignment time has been met (12 seconds) the designated cross section is exposed and selectively polymerized. Following the orientation phase, the entire area consisting of the orientation is polymerized using UV light. Multiple orientations can be achieved in single layer by repeating the process of orientation and polymerization. Once a layer is complete, a peel function lifts the build plate and allows for fresh resin to flow in between the previous layer and the resin container. This process is repeated until the part is complete. For example, the Boston skyline (**Supplementary Figure 2**) was printed in two steps. First, the regions oriented out-of-plane (dark regions) were aligned and subsequently polymerized. The remaining area of the print was then oriented in-plane (light regions) followed by polymerization. After parts completed the build process, they were rinsed in isopropyl alcohol and post-cured in a UV chamber (UVL-56, 6W, 365 nm) for 30 minutes followed by a heat treatment (90°C for 1 hr) to relieve any residual stresses. Printed parts have a maximum X-Y and Z resolution of 90 µm.

Tensile Testing. The mechanical properties of the cured composite resin were tested by printing sheets and subsequently punching out specimens using an ASTM-D638 IV die (Pioneer Dietics). All samples were tested on a universal testing machine (Instron 5960 Universal Testing Machine). Three primary reinforcement orientations were tested: parallel, perpendicular, and at an angle to the applied tensile load. Loading composites with 15% vol.

reinforcing particles aligned parallel to the load increased stiffness by more than 300%. Orienting particles perpendicular to the applied load increased stiffness by 200% vs. the pure polymer (**Supplementary Figure 3**).

Interface Analysis. Tensile tests were conducted on dogbones (10% volume fraction Al_2O_3) with reinforcement oriented in parallel, perpendicular, and at 45 degrees from the axis of the applied load. The fracture surfaces of each group demonstrate a shift in mode of failure from normal stress dominated fracture (parallel alignment) to shear dominated fracture (45 degrees) and cleavage (perpendicular). In the SEM images of the fracture surface (**Supplementary Figure 4**) for each orientation illustrate how the orientation of reinforcing particles affects crack propagation. Samples reinforced along the axis of the applied load (parallel alignment) demonstrated not only improved mechanical properties but visible signs of greater yielding. The crack propagates against platelet orientation with no true easy axis, and is therefore rough yet random. Conversely, samples reinforced at an angle demonstrated crack propagation congruent with the angle of the reinforcing particles, while samples reinforced orthogonal to the applied load demonstrated cleavage due to the crack propagating parallel to the platelets. To further corroborate that boundaries and interfaces do not serve as defects, tensile samples were printed with parallel alignment except for a small region. When tested, the failure was located at the center of the weaker region, rather than at the interface.

Mechanical testing of dogbones with circular defects. Osteon inspired samples were printed with a concentrically-reinforced hole in the center of a tensile dogbone specimen. Inspiration was drawn from the concentric plywood microstructure of collagen fibrils within osteons of mammalian cortical bone (**Figure 1g** in the main text). This reinforcement arrangement in nature results in a tailored, three-dimensional reinforcement that protects the harversian canal in the center of the osteon. When translated to the concentric reinforcement in a tensile specimen, the result is an isotropic mechanical response in which the mechanical properties are independent of the applied load. Samples with osteon-inspired orientations were compared against samples with reinforcement oriented parallel to the applied load (strong axis), perpendicular with the applied load (weak axis), and at various angles in between. It is important the note that each sample was printed with 8 discrete regions in the center to eliminate the interfacial regions as a source of discrepancy between each group of specimens. The orientation of the immediate regions surrounding the defect resulted in significant changes in mechanical properties as well as failure behavior. In particular, the crack path of the dogbones reinforced with particles oriented perpendicular to the load fail with a cleaved interface while less direct crack paths can be observed in specimens with parallel or osteon inspired reinforcement (**Supplementary Figure 5 and 6**). It is important to note that the samples did not delaminate at the interfaces between areas of different particle orientations.

Numerical methods to predict performance of reinforcing architecture. In the present study, we employed numerical methods to predict the performance of the reinforcing structures that encompassed the circular defects in the previous section. When the dogbones were subjected to a linear strain, such as from a tensile test, the specific reinforcement architecture that surrounded the defect strongly affected the local principal strains. To model these anisotropic material responses, we used COMSOL Multiphysics software. We imported the dogbone geometry with the circular defect into the software. We divided the region that surrounded the circular defect into the 8 equivalent shown in **Supplementary Figure 7** and consistent with our printing process described in the previous section. These 8 regions and the background region were each modeled as transversely isotropic materials. The elasticity stiffness matrix for transversely isotropic materials has five independent constants.

In the general case, the stress and strain are related with the elasticity stiffness matrix as follows:

$$\begin{bmatrix} \sigma_1 \\ \sigma_2 \\ \sigma_3 \\ \tau_{23} \\ \tau_{31} \\ \tau_{12} \end{bmatrix} = \begin{bmatrix} C_{11} & C_{12} & C_{13} & C_{14} & C_{15} & C_{16} \\ C_{21} & C_{22} & C_{23} & C_{24} & C_{25} & C_{26} \\ C_{31} & C_{32} & C_{33} & C_{34} & C_{35} & C_{36} \\ C_{41} & C_{42} & C_{43} & C_{44} & C_{45} & C_{46} \\ C_{51} & C_{52} & C_{53} & C_{54} & C_{55} & C_{56} \\ C_{61} & C_{62} & C_{63} & C_{64} & C_{65} & C_{66} \end{bmatrix} \begin{bmatrix} \varepsilon_1 \\ \varepsilon_2 \\ \varepsilon_3 \\ \gamma_{23} \\ \gamma_{31} \\ \gamma_{12} \end{bmatrix} \tag{4}$$

To establish the values for the constants in the elasticity matrix, we first consider the special case when the reinforcement is aligned at $\theta = 0°$. By assuming that the structure is only in a plane stress state, we can ignore contributions in the out-of-plane direction. This leads to the following elasticity stiffness matrix:

$$\begin{bmatrix} \sigma_1 \\ \sigma_2 \\ \tau_{12} \end{bmatrix} = \begin{bmatrix} C_{11} & C_{12} & 0 \\ C_{12} & C_{22} & 0 \\ 0 & 0 & C_{66} \end{bmatrix} \begin{bmatrix} \varepsilon_1 \\ \varepsilon_2 \\ \gamma_{12} \end{bmatrix} \tag{5}$$

In which:

$$C_{11} = \frac{E_1}{1 - \nu_{12}\nu_{21}} \tag{6}$$

$$C_{12} = \frac{\nu_{12}E_2}{1 - \nu_{12}\nu_{21}} = \frac{\nu_{21}E_1}{1 - \nu_{12}\nu_{21}} \tag{7}$$

$$C_{22} = \frac{E_2}{1 - \nu_{12}\nu_{21}} \tag{8}$$

$$C_{66} = G_{12} \tag{9}$$

For this special case, we have a strong axis with $E_1 = 900\ MPa$ and a weak axis with $E_2 = 690\ MPa$, as established in the tensile tests of monolithic samples presented in Figure 3 of the paper. We take the following values for the Poisson Ratios and the shear modulus: $\nu_{12} = 0.26$, $\nu_{21} = 0.20$, and $G_{12} = 370\ MPa$. These provide the initial elasticity stiffness matrix as follows:

$$[C] = \begin{bmatrix} 923\ MPa & 186\ MPa & 0 \\ 186\ MPa & 718\ MPa & 0 \\ 0 & 0 & 370\ MPa \end{bmatrix} \tag{10}$$

From this case, we can calculate the transformed stiffness tensor when we rotate the reinforcement at an angle of θ as follows[2]:

$$\bar{C}_{11} = C_{11}c^4 + C_{22}s^4 + (2C_{12} + 4C_{66})c^2s^2 \tag{11}$$

$$\bar{C}_{12} = C_{12}(c^4 + s^4) + (C_{11} + C_{22} - 4C_{66})c^2s^2 \tag{12}$$

$$\bar{C}_{22} = C_{11}s^4 + C_{22}c^4 + (2C_{12} + 4C_{66})c^2s^2 \tag{13}$$

$$\bar{C}_{16} = (C_{11} - C_{12} - 2C_{66})c^3s - (C_{22} - C_{12} - 2C_{66})cs^3 \tag{14}$$

$$\bar{C}_{26} = (C_{11} - C_{12} - 2C_{66})cs^3 - (C_{22} - C_{12} - 2C_{66})c^3s \tag{15}$$

$$\bar{C}_{16} = (C_{11} + C_{22} - 2C_{12} - 2C_{66})c^2s^2 + C_{66}(c^4 + s^4) \tag{16}$$

Where $c = \cos\theta$ and $s = \sin\theta$.

This methodology was employed to run the numerical simulations for conducting stress-strain analysis for various architectures. The architectures considered are listed in **Supplementary Table 1.**

Hardness Mapping. A rectilinear pattern (**Supplementary Figure 8**) was printed at 15% volume fraction alumina particles with a 22 x 22 x 3 mm (20 layers). Hardness mapping was conducted using Vickers micro-indentation. Indentations were made using a micro Vickers hardness tester (Phase II 900-390A) with an applied force of 0.98 N and 15 second dwell time. Indentations were made with a spacing of 1.5 mm, which is greater than 5 times the average diagonal length (approximately 200 microns). Sample thickness and test site spacing prevented indentations from affecting subsequent tests. The rectilinear pattern consisted of two areas (inner and outer) with an in-plane orientation and a center band with an out-of-plane orientation. Data points were collected (77 total) across the sample and can be viewed on the contour map in **Supplementary Figure 8**. Surface plots were generated using MatLab with a 1.5 mm grid and nearest neighbor interpolation in order to accurately represent the specimen.

Crack Steering. The ability to manipulate fracture behavior was further investigated by printing composite blocks with backgrounds of changing homogenous reinforcement orientation containing square islands of unique reinforcement orientations (**Supplementary Figure 9**). A small crack was initiated by hammering a razor blade into each sample. The samples were subsequently subjected to strain at 2 mm/minute. It was observed that the crack path could be altered by changing the reinforcement architecture within each specimen. For example, a pattern was printed where 4 mm x 4 mm islands were geometrically offset and reinforced against crack propagation (out-of-plane parallel to the stress), while the background was oriented parallel to the crack direction (out-of-plane perpendicular to the stress), (shown in **Supplementary Figure 9a**). The energy required for the crack to propagate through each reinforced island was greater than the energy required to propagate around it. Therefore, there was an obvious crack deflection. In another example, the islands instead were not geometrically offset and still

reinforced parallel to the crack direction (out-of-plane perpendicular to the stress), while the background was oriented against the crack direction (in-plane parallel to the stress) shown in **Supplementary Figure 9b**. In this case, the crack path is tortuous in the background and is driven to the weaker islands that fail in simple cleavage. Here, proper programming of the weak islands might lead to mechanisms that can increase the overall tortuosity of the crack path and therefore effectively enhance the fracture energy of these materials. In a third example, both the islands and the matrix phase were oriented in-plane parallel to the stress. A monolithic structure is produced and failure mechanisms are predictable (**Supplementary Figure 9c**). This system provides a novel method for measuring fracture energies of systems with complex reinforcement structures, and will be further investigated.

Bio-architectures in translation to architectural design – The Osteon of Cortical Bone. The structure of cortical bone has been long studied due to its impressive balance of strength, stiffness, and toughness. At the microscopic level, the mammalian cortical bone exhibits concentric plywood structures of lamellae-reinforced osteons[3]. It is believed that this reinforcement scheme arises from the need to protect the Haversian canals, which house cardiovasculature and parts of the nervous system[4]. Translating this structure to a concentric reinforcement orientation allows for reinforcement along any axis in the 2D plane. Samples with this concentric reinforcement orientation were printed in a hexagonal array, which each hole reinforced by eight discrete areas aligned tangentially to the center circle. The hierarchical orientation scheme can be observed (**Supplementary Figure 10**).

Bio-architectures in translation to architectural design – The Shell of the Abalone. The bi-layer structure of nacre was mimicked by printing a block (10% vol. magnetic particles) consisting of one in-plane reinforced region beneath an out of plane layer. SEM images of the cross-section obtained via freeze-fracture show not only good alignment, but a continuous interfacial region between phases (**Supplementary Figure 11**).

Bio-architectures in translation to architectural design – The Dactyl Club of the Peacock Mantis Shrimp. A 3D printed block (10% vol. magnetic particles) was printed with a cholesteric (helicoidal) reinforcement structure mimicking that of the peacock mantis shrimp. Each layer is rotated 45 degrees about the z-axis with respect to the subsequent layer. SEM images display solid interfacial bonding and proper alignment of each layer (**Supplementary Figure 12)**.

Controlling Optical Properties through particle orientation. In addition to mechanical properties, it was found that changing the orientation of the micro-particles also changed the way light interacts with the sample. For example, the darker areas in **Supplementary Figure 13a-b** are oriented out of plane, whereas the lighter regions are oriented

in-plane. Due to the low surface coating of iron-oxide on the surface of the alumina particles, platelets oriented in-plane expose primarily highly reflective alumina. This acts to scatter light within the sample, creating a lighter appearance. In contrast, the out of plane orientation allows for more of the light to penetrate the surface and be absorbed by the iron oxide on the surface of the alumina particles. Orienting particles at various angles can create a color-shift effect when tilting the sample (**Supplementary Figure 13c**). This is most apparent when comparing the angled orientations (135° and 45°). When the sample is tilted +45° and –45°, the color of each orientation on the sample is transposed. This technique may be further investigated for use in programming digital data into 3D printed materials.

Supplementary References:

1. Erb RM, Segmehl J, Schaffner M, Studart AR. Temporal response of magnetically labeled platelets under dynamic magnetic fields. *Soft Matter* **9**, 498-505 (2013).

2. Hull D, Clyne TW. *An Introduction to Composite Materials*. Cambridge University Press (1996).

3. Weiner S, Wagner HD. The material bone: Structure mechanical function relations. *Annu. Rev. Mater. Sci.* **28**, 271-298 (1998).

4. Wegst UGK, Bai H, Saiz E, Tomsia AP, Ritchie RO. Bioinspired structural materials. *Nat. Mater.* **14**, 23-36 (2015).
